# Disease Impact, Diagnostic Delay, and Unmet Medical Needs of Patients With Cholinergic Urticaria in German-Speaking Countries

**DOI:** 10.3389/falgy.2022.867227

**Published:** 2022-05-25

**Authors:** Sabine Altrichter, Emilia Mellerowicz, Dorothea Terhorst-Molawi, Eva Grekowitz, Karsten Weller, Marcus Maurer

**Affiliations:** ^1^Institute for Allergology, Charité – Universitätsmedizin Berlin, Corporate Member of Freie Universität Berlin and Humboldt-Universitätzu Berlin, Berlin, Germany; ^2^Department of Dermatology and Venerology, Kepler University Hospital, Linz, Austria; ^3^Fraunhofer Institute for Translational Medicine und Pharmacology ITMP, Allergology and Immunology, Berlin, Germany; ^4^Clinical Physiology/Nutritional Medicine, Charité – Universitätsmedizin Berlin, Corporate Member of Freie Universität Berlin and Humboldt-Universität zu Berlin, Berlin, Germany

**Keywords:** cholinergic urticaria, unmet medical needs, wheals, angioedema, hives, mast cells

## Abstract

**Background:**

Cholinergic urticaria (CholU) is a common type of chronic inducible urticaria. Little is known about the burden of the disease and its unmet medical needs.

**Aim:**

To characterize the unmet medical needs of patients with CholU.

**Methods:**

Patients with CholU (*n* = 111) took part in a German online survey that assessed their symptoms, diagnostic delay, impact on daily life, quality of life (QoL), and their experience with physician care.

**Results:**

Virtually all patients reported typical signs and symptoms of CholU, i.e., whealing (93.7%) and itching (91.9%), in response to typical trigger situations, such as physical activity, passive warming, or stress. Despite this, patients reported a marked diagnostic delay of 30.2 months (range from 0 to 279 months). Only 38% of the patients received a blood examination, and only 16% underwent provocation testing for diagnosing CholU, as recommended by the international guidelines. Physician contacts were common, but patient satisfaction with their disease management was low. In total, 90.1% of the patients stated to have an uncontrolled disease, resulting in a strong impact on their everyday activities, sleep, and QoL.

**Conclusion:**

Patients with CholU exhibit many important unmet needs, and improvement in the diagnostic workup and patient care is needed, as are better treatment options.

## Introduction

Cholinergic urticaria (CholU) is a frequent skin disorder that manifests with pinpoint-sized itchy wheals and, in up to 50% of patients, with angioedema (1). CholU is a form of chronic inducible urticaria and is triggered by sweating, e.g., due to passive warming of the body or physical activity (2–4). Disease severity can range from mild symptoms in severe trigger situations, such as extensive sporting, that can be controlled by avoiding these triggers up to extensive outbreaks in everyday situations, such as climbing stairs.

Cholinergic urticaria, i.e., all forms of chronic urticaria, shows spontaneous remission, usually after several years. The aim of treatment was to achieve complete disease control either by pieces of advice in their daily life (e.g., avoiding trigger situations, using refractory phases after severe outbreaks etc.) or by providing patients with medication that completely controls the disease until this happens. Treatments for CholU, according to the international guideline for urticaria (5), include second-generation H1-Antihistamines at standard dose, with updosing to up to 4-fold in patients with an insufficient response. When antihistamines fail, treatment with omalizumab or ciclosporin is recommended (5), but these are not licensed for the use in CholU. We recently reported on the outcomes of real-life therapy for patients with CholU in German-speaking countries and demonstrated that current treatment options often fail and better therapies are needed (6, 7).

Cholinergic urticaria has a high impact on the quality of life (QoL) of the patients (4, 8) but many aspects of the disease remain poorly understood and the impact of having CholU is often underestimated. Some of the major unanswered questions on CholU are as follows: How heterogeneous are patients with CholU in their clinical manifestations and relevant triggers? How much does the disease affect patients in their everyday life and QoL? How long does it take for patients with CholU to get diagnosed? Which physicians treat patients with CholU and what diagnostic measures do they use? How many patients are in medical care and how satisfied are CholU patients with their doctor-patient relationship? Although symptoms of CholU can affect up to 20% of the population in the age group of 26–28 years (9), little is known about these questions. To address this gap of knowledge and to understand more about the unmet medical needs and the burden of CholU, we performed an online study in German-speaking countries.

## Materials and Methods

### Study Design and Participants

We analyzed 111 patients who took part in an online survey study on CholU in German-speaking countries, performed by the Charité Urticaria Center of Reference and Excellence [UCARE, (10)], the Urtikaria Netzwerk e.V., and the Urtikaria Netzwerk Berlin-Brandenburg from May 2016 to August 2017. This study was approved by the Ethics Committee of the Charité - Universitätsmedizin Berlin (#EA1/241/15) and registered in the German Clinical Trials Register (DRKS-ID: DRKS00012387).

Patients from Germany, Switzerland, and Austria participated anonymously in the survey. The patients had to be at least 18 and had to confirm their consent before starting the survey.

In total 197 patients participated in the online survey. Of these, 111 had CholU that was confirmed by a physician and stated suitable trigger factors, and only these patients were included in our study.

The questionnaire was divided into five parts with questions on demographics, the course of the disease, impact on work and daily life, patient-doctor relationship, and treatment.

In the present report, we focused on the unmet medical needs and the disease burden of patients with CholU. Results of the online survey regarding the real-life treatment situation of patients with CholU in German-speaking countries have previously been reported (6).

In our survey, we included the Urticaria Control Test (UCT) and the Cholinergic Urticaria Quality-of-Life Questionnaire (CholU-Qol). We used the UCT (Moxie, Berlin, Germany) to assess disease control in our patients (11). The UCT has four questions with five answer options each, with a score between 0 and 4 assigned to every answer option. To calculate the UCT total score, the scores for all four questions were summed up. Accordingly, the minimum and maximum UCT scores were 0 and 16, respectively, with 16 points indicating complete disease control and scores below 12 indicating poorly controlled disease.

The CholU-QoL [Moxie, Berlin, Germany; (12)] is a recently developed patient-reported outcome measure for assessing CholU-specific QoL impairment. The CholU-QoL has 28 questions and a five domain structure (“symptoms,” “functional life,” “social interaction,” “therapy,” and “emotions”). The CholU-QoL is meant to be evaluated by using its five individual domains (profile instrument) but it can also be used to determine a total score (index instrument). Points from 0 to 4 were given for the response options: not at all/no treatment, somewhat, moderately, much, or very much, respectively. The CholU-QoL domain scores and the CholU-QoL total scores are calculated by using the following formula: (*Σ* items/max *Σ* items) x 100. The linear transformation of raw scores results in minimal and the highest possible scale and total scores of 0 and 100, respectively.

The total score was not computed when >20% of the items (>5 items) were missing. The domain scores “symptoms,” “functional life,” “social interaction,” and “emotions” were not computed when more than 25% of the items were missing in the respective domain. The domain score “therapy” was not computed when >50% of the items were missing.

Missing hours from work in the last week was evaluated, and subjective work impairment in the last week on a scale of 0 (no impairment) to 10 was also evaluated (no work possible).

### Statistical Analyses

Statistical analyses were performed using IBM SPSS Statistics version 23. Graphs were made with GraphPad Prism Version 6.0 and Excel Version2016. *p* < 0.05 was used to determine statistical significance.

## Results

Patients participating in the online survey, on average, were 38.7 years old (range: 18–78 years), and 76.6% were women.

### Clinical Manifestation and Triggers

The majority of patients reported wheals (93.7%), pruritus (91.9%), or both as their main manifestation of CholU. One in three patients (35.1%) reported angioedema. Circulation problems and dizziness occurred in 38.7 and 18.9% of patients with CholU, respectively. Only 4 patients (3.6%) experienced unconsciousness. Most of the patients (53%) stated a typical symptom duration between 30 min and 2 h. Only a few reported shorter durations (4%) and several times more than 2 h (39%).

As for trigger factors, most patients (86.5%) named the physical activity. Other common triggers included taking a warm bath, emotional stress, and feeling agitated, which were reported by 54.1, 50.5, and 47.7% of patients, respectively. Less common triggers, in 38.7, 20.7, and 13.5% of patients, respectively, were showering, spicy food, and hot food or drinks.

### Diagnostic Delay and Physician-Patient Interaction

The median time between first signs and symptoms of CholU and receiving the diagnosis was 30.2 months, with a wide range of 0–279 months. In more than 60% of the patients, the diagnosis was given at least a year after the onset of the symptoms or later.

Most patients (43.2%) first consulted a dermatologist followed by a general practitioner/family physician (37.8%). Very few patients initially turned to an emergency room (3.6%), outpatient consultation of a clinic (3.6%), or a specialized urticaria clinic (2.7%).

Upon their first encounter with a physician, more than one-third of patients with CholU (38%) received a blood examination, but only 16% underwent provocation testing, the guideline-recommended approach for confirming chronic inducible urticaria (CIndU) that includes CholU (see Table 1).

**Table 1:** Patient-reported diagnostic workup, topics of discussion, and treatments upon the first presentation (multiple answers in each category were possible).

	**1st physician**	**2nd physician**	**Current physician**
Dermatologist	48/111, 43.2%	44/82, 53.7%	31/111, 27.9%
Family Doctor/ general practitioner	42/111, 37.8%	7/82, 8.5%	20/111, 18.0%
Special consultation hours for urticaria patients	3/111, 2.7%	11/82, 13.4%	6/111, 5.4%
Ambulant consultation hour of a clinic	4/111, 3.6%	6/82, 7.3%	7/111, 6.3%
Medical on-call service	0/111, 0%	1/82, 1.2%	-
Emergency room	4/111, 3.6%	3/82, 3.7%	-
Other	8/111, 7.2%	8/82, 9.8%	5/111, 4.5%
None	-	-	38/111, 34.2%
No data	2/111, 1.8%	2/82, 2.4%	4/111, 3.6%
**Diagnostics**			
Blood-examination	42/111, 37.8%	31/82, 37.8%	28/69, 40.6%
Allergy testing	30/111, 27.0%	28/82, 34.1%	17/69, 24.6%
Full Body examination	27/111, 24.3%	29/82, 35.4%	22/69, 31.9%
Provocation-testing	18/111, 16.2%	14/82, 17.1%	13/69, 18.8%
Other examinations	16/111, 14.4%	10/82, 12.2%	11/69, 15.9%
**Topics of discussion**			
Discussion about possible causes of the CholU	42/111, 37.8%	40/82, 48.8%	30/69, 43.5%
Discussion about treatment- possibilities	37/111, 33.3%	35/82, 42.7%	31/69, 44.9%
Discussion about possible progress of the CholU	32/111, 28.8%	33/82, 40.2%	33/69, 47.8%
**Treatment**			
Recommended treatment with a drug	46/111, 41.4%	38/82, 46.3%	37/69, 53.6%
Immediate application of a drug	34/111, 30.6%	17/82, 20.7%	15/69, 21.7%
Other^*^	18/111, 16.2%	11/82, 13.4%	11/69, 16.0%
No data on consultation	2/111, 1.8%	3/82, 3.7%	5/69, 7.2%
**Treatment satisfaction**			
Not at all satisfied	36/111, 32,4%	18/82, 22%	8/69, 11,6%
Not very satisfied	38/111, 34,2%	28/82, 34,1%	25/69, 36,2%
Satisfied	28/111, 25,2%	23/82, 28%	19/69, 27,5%
Very satisfied	5/111, 4,5%	9/82, 11%	8/69, 11,6%
No data	4/111, 3,6%	4/82, 4,9%	9/69, 13%

* *Including urine tests, stool sampling and analysis, test for scabies, dental X-ray, CT of the head, ultrasound of internal organs, colonoscopy, and lung function test, referral to the specialized center*.

Of note, most patients (82 of the 111; 73.9%) visited a second physician, and this was a dermatologist in half of the cases (53.7), a specialized urticaria clinic (13.4%), and a general practitioner/family physician (8%). Most patients (45%) did so on their own, whereas 44% were referred by their treating physician.

Even after the second visit, more often in a more specialized setting, the stated diagnostic procedures and treatment choices did not change dramatically. Again 38% of (31 of 82) patients with CholU received a blood examination and 14 out of 82 patients underwent provocation testing (17%).

Of the patients who provided information on their level of satisfaction with their treatment of the first physician, 67% reported that they were not or only somewhat satisfied, whereas 30% of patients were satisfied or very satisfied.

About 30–40% of the patients were very satisfied or satisfied with their first or second physician encounter, whereas 24–30% of the patients were not at all satisfied and 32–35% of patients were only slightly satisfied.

One of three patients (34.2%) were currently not in physician care for their CholU (see Table 1).

### Disease Activity and Control

**Figure 1:**
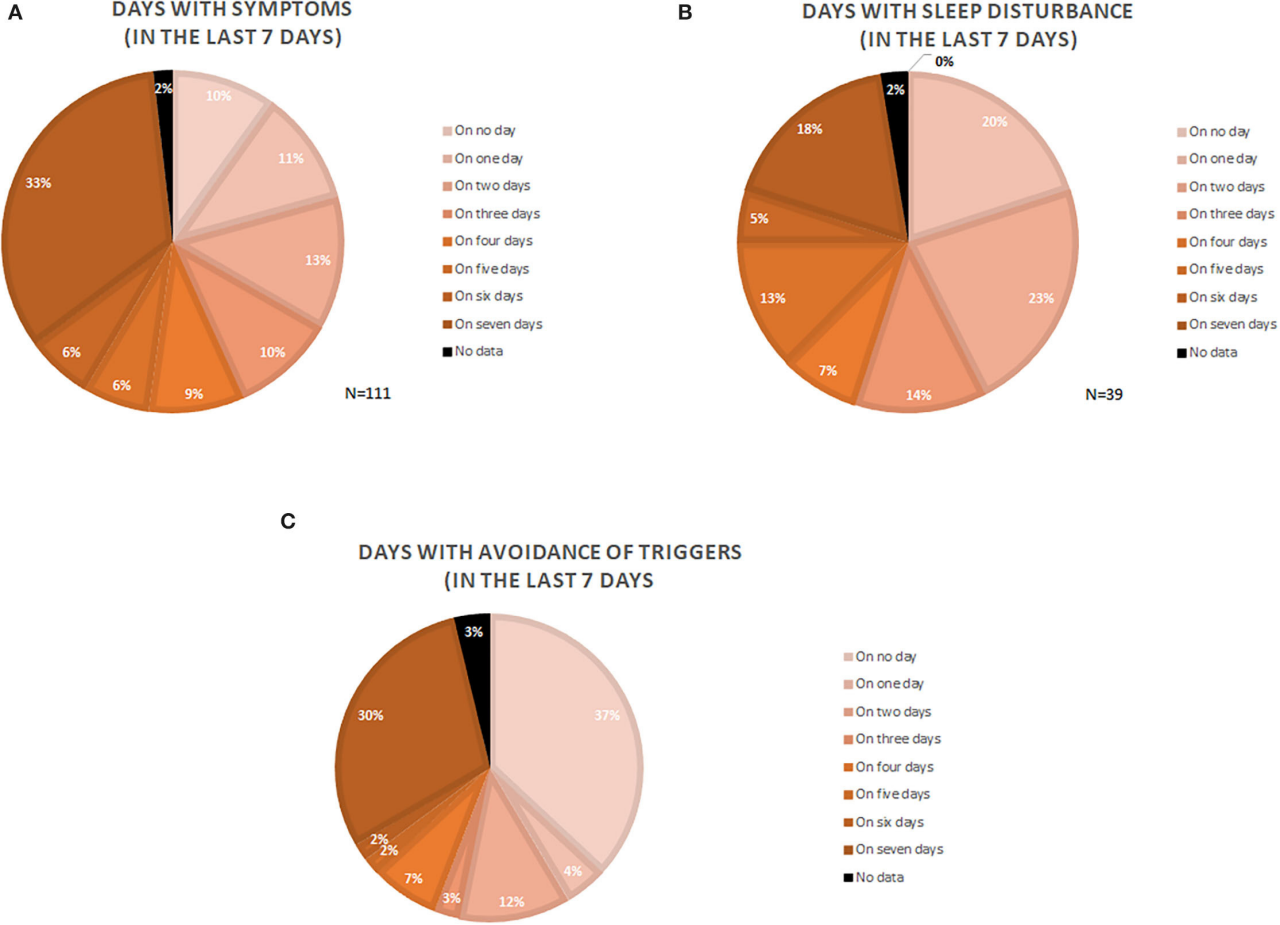
Depiction of symptomatic days **(A)**, days with sleep disturbance **(B)**, and days where patients actively avoided trigger situations **(C)**. Numbers are given as the percentage of the analyzed patients (*N* = number of patients).

One-third of the patients with CholU (33.3%) reported daily symptoms within the last 7 days. More than half (55%) had symptoms on more than 3 days of the week, and only 10% of patients had no symptoms during the last 7 days (Figure 1A).

One of three patients (35.1%) had trouble sleeping due to their CholU, and one of five patients (18%) had sleep problems due to CholU on all nights of the week (Figure 1B).

Every third patient (29.7%) avoided situations that could trigger symptoms on all of the 7 days of the last week, whereas 36.9% of the patients did not do this at all. More than half of the patients (55%) avoided trigger situations on 2 or more days of the week (Figure 1C).

### Impact on Daily Activities and QoL

**Figure 2:**
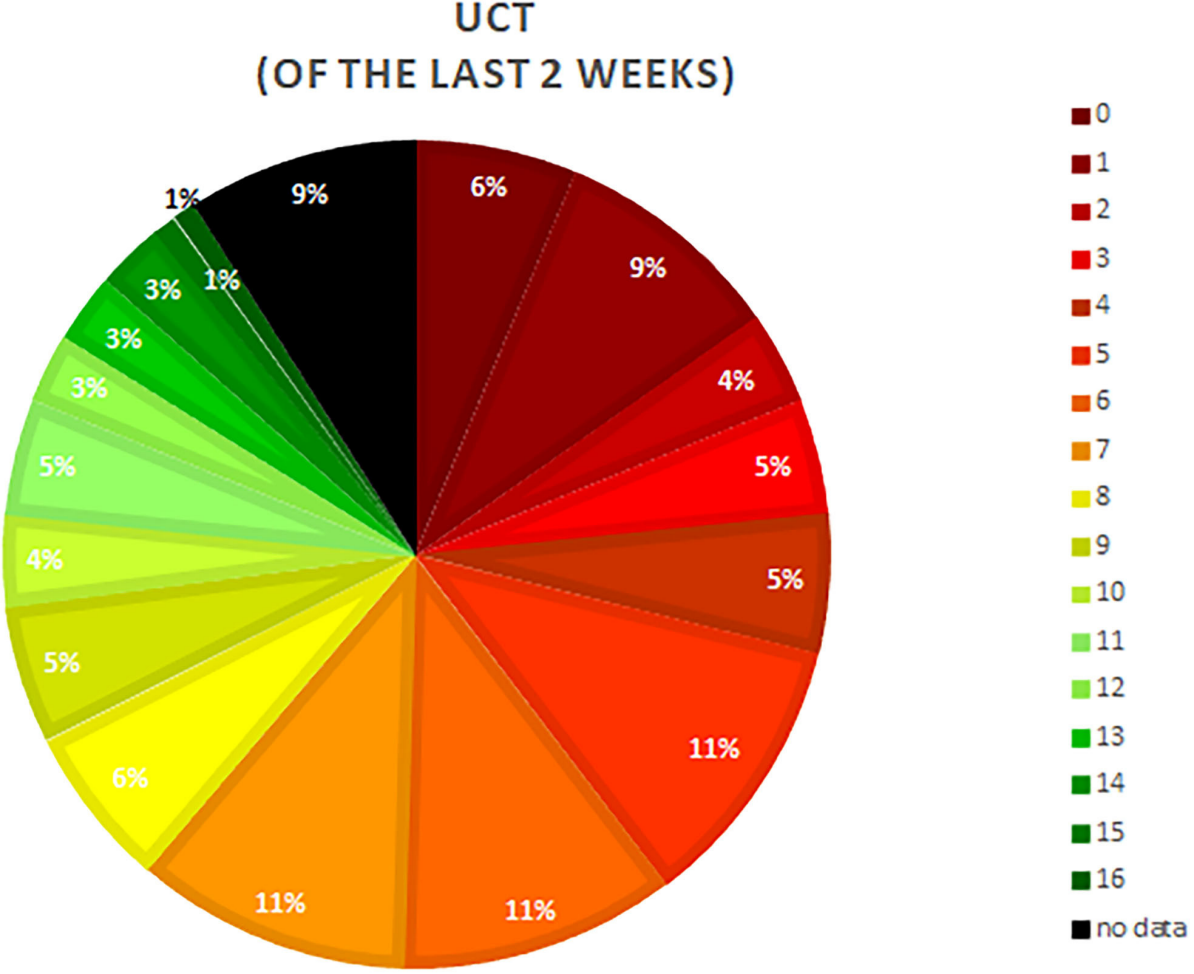
Depiction of the distribution of the Urticaria Control Test (UCT) point results. UCT scores of more than 12 points indicate sufficient disease control. The lower the achieved points the less controlled the disease was at the time point of assessment.

Nine of 10 patients (90.1%) had poorly controlled disease as reflected by a UCT score of 11 or less, and only 1% reported complete control (UCT = 16, Figure 2).

In our study, 45, 31, and 23% of patients, respectively, reported that their overall life quality suffered much or very much, moderately, and somewhat or not at all, during the last 4 weeks because of their CholU. As assessed by the use of the disease-specific QoL questionnaire CholU-QoL, patients, on average, showed markedly impaired QoL as reflected by a mean (±SD) CholU-QoL score of 47.5 ± 13.5. The highest impact of CholU was seen in the social interaction domain (63.1 ± 25.2), followed by the domains therapy (63.8 ± 19.2) and functioning (61.6 ± 21.5).

**Figure 3:**
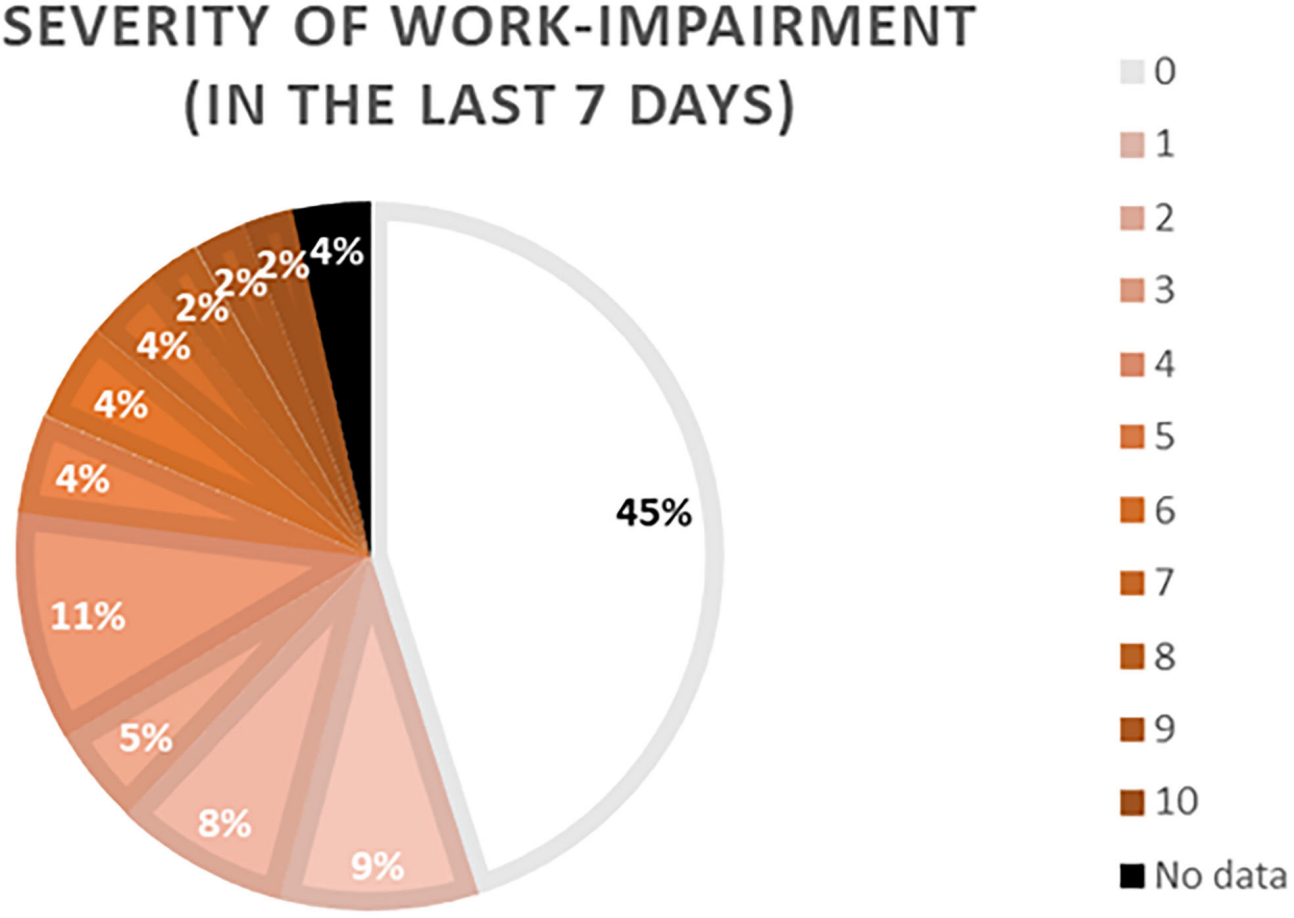
Depiction of the patient's rating of their severity of work impairment in the last 7 days due to cholinergic urticaria (CholU) symptoms. Patients could rate on a 10-point Likert scale ranging from 0 (no impairment) to 10 (max. impairment).

Of the 111 patients, 88 (79.3%) reported to have a job. More than 50% of these patients stated that their productivity was impaired to some extent (see Figure 3). Moreover, 27% of the professional working patients reported that they missed work in the last week due to CholU symptoms (mean 14.7 h ± 14.0; range 1–48 h).

## Discussion

This study, the first on the unmet needs of patients with CholU in Germany, Austria, and Switzerland, demonstrates that patients with CholU face long delays in diagnosis, insufficient diagnostic workup, and medical treatment that many find unsatisfactory and high levels of disease activity, uncontrolled disease, and impairment of QoL.

As expected, most patients with CholU experience wheals and pruritus in response to relevant triggers, about one-third have angioedema. Severe systemic reactions that include loss of consciousness are extremely rare. All of this is in line with what has previously been reported in other patient cohorts (1, 13–15). Mild systemic signs and symptoms, such as dizziness and circulatory problems, appear to be more common than commonly held. More than half of the patients (57%) reported symptom duration of up to 2 h. A large proportion (39%) also reported symptoms of longer durations, that fit to severely affected patients that experience angioedema, since they tend to last longer. However, we cannot rule out that patients who either have a heat aggravating chronic spontaneous urticaria or patients who have both CholU and chronic spontaneous urticaria might have been included in the survey.

Physical exercise is the most common trigger of signs and symptoms comes as no surprise (2) and neither does the finding that passive warming is a frequent trigger (16). What is interesting is that emotional stress or feeling agitated, in about half of patients each, is sufficient to elicit CholU symptom development. Emotional stress and agitation had previously been reported as triggers in CholU, albeit mostly anecdotally (17, 18).

Three-quarters of the analyzed study participants were women. This unexpectedly high number of women could point to a higher disease burden in women that typically motivates patients to participate in such surveys and/or to a common fact that women incline toward earlier seek for medical advice/treatment (19) resulting in a physician made the diagnosis, which was an inclusion criterion.

The reason for concern is our finding that the average time for patients with CholU to receive the correct diagnosis is 2.5 years. This is concerning because not knowing what disease is responsible for their signs and symptoms can be a burden for patients and it often delays effective treatment. The long delay in diagnosis is also somewhat of a surprise since CholU, with a good history and provocation testing, is relatively easy to diagnose and many patients were first seen by a specialist (dermatologist). One reason for this long delay in diagnosis may be the fact that only one of six patients was assessed by provocation testing, the guideline-recommended test of choice in CholU (2, 5). Provocation testing for CholU is straightforward and easy to perform (20). Clearly, there is a need to increase awareness and knowledge of CholU in the physician community, especially on the diagnostic workup.

Overall, patients consulted four physicians on average because of their CholU and most patients were not satisfied with their medical care. This explains why one-third of the patients did not currently work with a physician to manage their CholU. Only about one-third of the patients were currently in specialist/dermatologist care. This explains, at least in part, our previously reported finding that half of the patients with CholU do not currently receive treatment for their condition (6).

Nine of ten patients with CholU reported poor control of their urticaria, and two reasons for this are likely. First, many patients do not receive treatment or receive treatment that does not help them control their CholU. Patients with CholU who do not respond to a standard-dosed antihistamine can benefit from updosing (21, 22), but this is not done in the conditions of many patients (6). Second, treatment options for CholU are limited. Omalizumab has been shown to benefit many patients with CholU who do respond to antihistamines at standard or high doses (23–26). However, omalizumab is off label for CIndU that includes CholU and only licensed for the use in patients with spontaneous forms of chronic urticaria. Moreover, at the time of the survey, omalizumab was a more novel treatment for patients with chronic urticaria. Of note, treatment recommendations aside from drugs (e.g., trying repetitive exercise to induce a refractory state, etc.) were only discussed with up to 16% of the patients. In our experience, such treatments can work in some patients but do not work in others and are hard to continue on a regular base. Clearly, more effective treatment options are needed for CholU, and several new therapeutics are currently in clinical development, such as lirentelimab [a mast cell-silencing anti-SIGLEC8 (27)], ligelizumab [an anti-immunoglobulin E [IgE] (28)], CDX-0159 [a mast cell-depleting anti-KIT (29)], and LEO 152020 [a histamine receptor 4 antagonist (30)].

The need for effective treatment, in patients with more severe symptoms of CholU, is high. Half of the patients experience the signs and symptoms of their CholU 5 or more days per week. One-third of patients with CholU proactively avoid trigger situations, which restrains their social activities and results in a reduced QoL. Most patients report their QoL to be moderately, much, or very much affected by their CholU. In addition, the professional productivity was impaired in more than half of the patients, resulting in an economic burden of the disease.

Limitations of this report include that data were obtained by an online survey, i.e., without verification by treating physicians. To minimize the possibility that some of the patients who participated did not have CholU, we only evaluated the responses of patients who indicated suitable trigger situations and who stated that their CholU was physician diagnosed. However, we cannot rule out that patients with other forms of urticaria (e.g., heat aggravated chronic spontaneous urticaria or with combinations of CholU and chronic spontaneous urticaria) were included. It is possible that there is a higher probability of patients with a higher disease burden and non-satisfactory treatment to participate in such an online survey, leading to an overestimation of disease burden. Moreover, we lack information about the compliance of patients with their treatment and physician advice, which could also be a reason for unsatisfactory disease control.

In summary, this report highlights the high need for better awareness and knowledge among physicians who treat patients with CholU that include specialists, such as dermatologists. The need for treatment is clearly being underestimated, and this may be because of a lack of understanding of the impact CholU has on patients. Urticaria specialists need to educate physician communities on the diagnostic workup, monitoring, and management of CholU, and the Undergraduate Creative Activities and Research Experience (UCARE) LevelUp program can help with this. Several new treatments for CholU are underway, and patients should be encouraged to participate in the ongoing clinical trials. In addition, prospective studies and further research on CholU are needed to identify and characterize pathogenic drivers and to help with the development of further treatment options.

## Data Availability Statement

The raw data supporting the conclusions of this article will be made available by the authors, without undue reservation.

## Ethics Statement

The studies involving human participants were reviewed and approved by local Ethic Comittee of Charite - Universitätsmedizin Berlin, Berlin, Germany. Written informed consent for participation was not required for this study in accordance with the national legislation and the institutional requirements.

## Author Contributions

SA conducted the study and drafted the manuscript. EM was involved in data management and statistical analysis. DT-M supported with logistics and was involved with proofreading of the manuscript. EG supported patient recruitment. KW was involved in the questionnaire development, data cleaning, and statistical analysis. MM was the overall project lead, gave continuous project support, and was involved with proofreading of the manuscript. All authors contributed to the article and approved the submitted version.

## Conflict of Interest

KW is a speaker and advisor and has received research funding from Biocryst, CSL Behring, Dr. Pfleger, FAES, Moxie, Novartis, Shire/Takeda, and Uriach. MM is a speaker and advisor and has received research funding from Allakos, Amgen, Aralez, ArgenX, AstraZeneca, Celldex, Centogene, CSL Behring, FAES, Genentech, GIInnovation, GSK, Innate Pharma, Kyowa Kirin, Leo Pharma, Lilly, Menarini, Moxie, Novartis, Roche, Sanofi/Regeneron, Third HarmonicBio, UCB, and Uriach. SA has been a speaker and advisor and has conducted studies for Astra Zeneca, Allakos, GSK, LeoPharma, Lilly, Moxie, Novartis, Thermo Fisher, and Sanofi. DT-M has been an advisor and has received research funding from Moxie, Novartis, Celldex, and Sanofi. The remaining authors declare that the research was conducted in the absence of any commercial or financial relationships that could be construed as a potential conflict of interest.

## Publisher's Note

All claims expressed in this article are solely those of the authors and do not necessarily represent those of their affiliated organizations, or those of the publisher, the editors and the reviewers. Any product that may be evaluated in this article, or claim that may be made by its manufacturer, is not guaranteed or endorsed by the publisher.

## Abbreviations

CholU: Cholinergic urticaria.

## References

[1] Mellerowicz EJ Asady A Maurer M Altrichter S. Angioedema frequently occurs in cholinergic urticaria. J Allergy Clin Immunol Pract. (2019) 7:1355–7. 10.1016/j.jaip.2018.10.013 30368007

[2] Magerl M Altrichter S Borzova E Gimenez-Arnau A Grattan CE Lawlor F. The definition, diagnostic testing, and management of chronic inducible urticarias - The EAACI/GA(2) LEN/EDF/UNEV consensus recommendations 2016 update and revision. Allergy. (2016) 71:780–802. 10.1111/all.12884 26991006

[3] Altrichter S Koch K Church MK Maurer M. Atopic predisposition in cholinergic urticaria patients and its implications. J Eur Acad Dermatol Venereol. (2016) 30:2060–5. 10.1111/jdv.13765 27324252

[4] Asady A Ruft J Ellrich A Hawro T Maurer M Altrichter S. Cholinergic urticaria patients of different age groups have distinct features. Clin Exp Allergy. (2017) 47:1609–14. 10.1111/cea.13023 28873238

[5] Zuberbier T Aberer W Asero R Bindslev-Jensen C Brzoza Z Canonica GW. The EAACI/GA(2) LEN/EDF/WAO Guideline for the definition, classification, diagnosis, and management of urticaria: the 2013 revision and update. Allergy. (2014) 69:868–87. 10.1111/all.12313 24785199

[6] Mellerowicz E Weller K Zuberbier T Maurer M Altrichter S. Real-life treatment of patients with cholinergic urticaria in German-speaking countries. J Dtsch Dermatol Ges. (2019) 17:1141–7. 10.1111/ddg.13979 31765087

[7] Maurer M Fluhr JW Khan DA. How to approach chronic inducible urticaria. J Allergy Clin Immunol Pract. (2018) 6:1119–30. 10.1016/j.jaip.2018.03.007 30033913

[8] Poon E Seed PT Greaves MW Kobza-Black A. The extent and nature of disability in different urticarial conditions. Br J Dermatol. (1999) 140:667–71. 10.1046/j.1365-2133.1999.02767.x 10233318

[9] Zuberbier T Althaus C Chantraine-Hess S Czarnetzki BM. Prevalence of cholinergic urticaria in young adults. J Am Acad Dermatol. (1994) 31:978–81. 10.1016/S0190-9622(94)70267-5 7962780

[10] Maurer M Metz M Bindslev-Jensen C Bousquet J Canonica GW Church MK. Definition, aims, and implementation of GA(2) LEN Urticaria Centers of Reference and Excellence. Allergy. (2016) 71:1210–8. 10.1111/all.12901 27038243

[11] Ohanyan T Schoepke N Bolukbasi B Metz M Hawro T Zuberbier T. Responsiveness and minimal important difference of the urticaria control test. J Allergy Clin Immunol. (2017):140 1710–3. 10.1016/j.jaci.2017.04.050 28625805

[12] Ruft J Asady A Staubach P Casale T Sussmann G Zuberbier T. Development and validation of the cholinergic urticaria quality-of-life questionnaire (CholU-QoL). Clin Exp Allergy. (2018) 48:433–44. 10.1111/cea.13102 29369455

[13] Iijima S Kojo K Takayama N Hiragun M Kan T Hide M. Case of cholinergic urticaria accompanied by anaphylaxis. J Dermatol. (2017) 44:1291–4. 10.1111/1346-8138.13951 28665007

[14] Rujitharanawong C Tuchinda P Chularojanamontri L Chanchaemsri N Kulthanan K. Cholinergic Urticaria: clinical presentation and natural history in a tropical country. Biomed Res Int. (2020) 2020:7301652. 10.1155/2020/7301652 32596363 PMC7273400

[15] Seo JH Kwon JW. Epidemiology of urticaria including physical urticaria and angioedema in Korea. Korean J Intern Med. (2019) 34:418–25. 10.3904/kjim.2017.203 29742892 PMC6406086

[16] Illig L Paul E Bruck K Schwennicke HP. Experimental investigations on the trigger mechanism of the generalized type of heat and cold urticaria by means of a climatic chamber. Acta Derm Venereol. (1980) 60:373–80. 10.2340/0001555560373380 6162306

[17] Illig L. [On the pathogenesis of cholinergic urticaria. I Clinical observations and histological studies]. Arch Klin Exp Dermatol. (1967) 229:231–47. 10.1007/BF00502986 5593413

[18] Pichler WJ Pichler CE Helbing A. [Exertion-induced anaphylaxis]. Schweiz Med Wochenschr. (1987) 117:9–16. 3810106

[19] Cornally N McCarthy G. Help-seeking behaviour for the treatment of chronic pain. Br J Community Nurs. (2011) 16:90–8. 10.12968/bjcn.2011.16.2.90 21378675

[20] Altrichter S Salow J Ardelean E Church MK Werner A Maurer M. Development of a standardized pulse-controlled ergometry test for diagnosing and investigating cholinergic urticaria. J Dermatol Sci. (2014) 75:88–93. 10.1016/j.jdermsci.2014.04.007 24837213

[21] Koch K Weller K Werner A Maurer M Altrichter S. Antihistamine updosing reduces disease activity in patients with difficult-to-treat cholinergic urticaria. J Allergy Clin Immunol. (2016) 138:1483–5. 10.1016/j.jaci.2016.05.026 27496595

[22] Zuberbier T Munzberger C Haustein U Trippas E Burtin B Mariz SD. Double-blind crossover study of high-dose cetirizine in cholinergic urticaria. Dermatology. (1996) 193:324–7. 10.1159/000246281 8993958

[23] Maurer M Metz M Brehler R Hillen U Jakob T Mahler V. Magerl, Omalizumab treatment in patients with chronic inducible urticaria: a systematic review of published evidence. J Allergy Clin Immunol. (2018) 141:638–49. 10.1016/j.jaci.2017.06.032 28751232

[24] Altrichter S Chuamanochan M Knoth H Asady A Ohanyan T Metz M. Real-life treatment of cholinergic urticaria with omalizumab. J Allergy Clin Immunol. (2019) 143:788–91. 10.1016/j.jaci.2018.08.050 30312709

[25] Metz M Altrichter S Ardelean E Kessler B Krause K Magerl M. Anti-immunoglobulin E treatment of patients with recalcitrant physical urticaria. Int Arch Allergy Immunol. (2011) 154:177–80. 10.1159/000320233 20733327

[26] Yu M Terhorst-Molawi D Altrichter S Hawro T Chen YD Liu BX, Omalizumab in chronic inducible urticaria: a real-life study of efficacy, safety, predictors of treatment outcome and time to response. Clin Exp Allergy. (2021) 51:730–4. 10.1111/cea.13838 33522024

[27] Altrichter S Staubach P Pasha M Singh B Chang AT Bernstein JA. An open-label, proof-of-concept study of lirentelimab for antihistamine-resistant chronic spontaneous and inducible urticaria. J Allergy Clin Immunol. (2021). S0091-6749(21)02682-8. 10.1016/j.jaci.2021.12.772. [Epub ahead of print]. 34954198

[28] Maurer M Gimenez-Arnau AM Sussman G Metz M Baker DR Bauer AJ, Ligelizumab for chronic spontaneous urticaria. N Engl J Med. (2019) 381:1321–32. 10.1056/NEJMoa1900408 31577874

[29] Maurer M Khan DA Elieh Ali Komi D Kaplan AP. Biologics for the use in chronic spontaneous urticaria: when and which. J Allergy Clin Immunol Pract. (2021) 9:1067–78. 10.1016/j.jaip.2020.11.043 33685605

[30] T. Bieber. Atopic dermatitis: an expanding therapeutic pipeline for a complex disease. Nat Rev Drug Discov. (2022) 21:21–40. 10.1038/s41573-021-00266-6 34417579 PMC8377708

