# Supplementary figures and images for: Disease Impact, Diagnostic Delay, and Unmet Medical Needs of Patients With Cholinergic Urticaria in German-Speaking Countries
Source: Front Allergy. 2022 May 25;3:867227. doi: 10.3389/falgy.2022.867227 (PMC9234879; doi:10.3389/falgy.2022.867227)

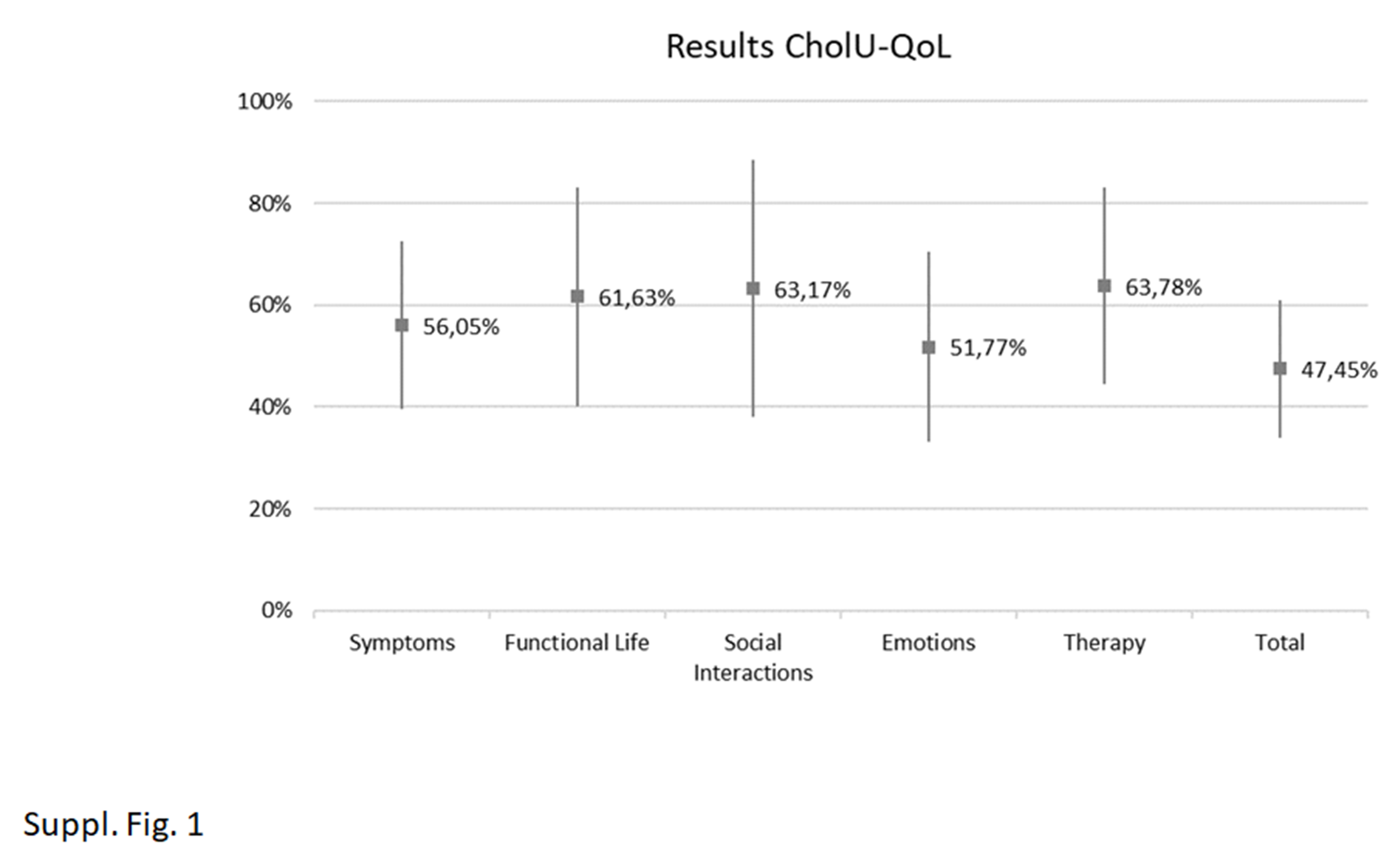

Supplement: Supplementary file 1 [file Image_1.tif]
